# Ribose 5-phosphate isomerase inhibits LC3 processing and basal autophagy

**DOI:** 10.1016/j.cellsig.2016.06.015

**Published:** 2016-09

**Authors:** Jacob Heintze, Joana R. Costa, Melanie Weber, Robin Ketteler

**Affiliations:** MRC Laboratory for Molecular Cell Biology, University College London, London, UK

**Keywords:** RPIA, Autophagy, CRISPR, Cas9, Pentose phosphate pathway, shRNA

## Abstract

Autophagy and cellular metabolism are tightly linked processes, but how individual metabolic enzymes regulate the process of autophagy is not well understood. This study implicates ribose-5-phosphate isomerase (RPIA), a key regulator of the pentose phosphate pathway, in the control of autophagy. We used a dual gene deletion strategy, combining shRNA-mediated knockdown studies with CRISPR/Cas9 genome editing. Knockdown of RPIA by shRNA or genomic deletion by CRISPR/Cas9 genome editing, results in an increase of ATG4B-mediated LC3 processing and in the appearance of LC3-positive autophagosomes in cells. Increased LC3 processing upon knockdown of RPIA can be reversed by treatment with the antioxidant *N*-acetyl cysteine. The results are consistent with a model in which RPIA suppresses autophagy and LC3 processing by modulation of redox signaling.

## Highlights

• Ribose-5-phosphate isomerase links autophagy with the pentose phosphate pathway.
• Generation of a CRISPR/Cas9 genome edited RPIA knockout cell line
• RPIA isomerase suppresses cellular LC3 processing and autophagosome formation.

## Graphical abstract

**Figure:**
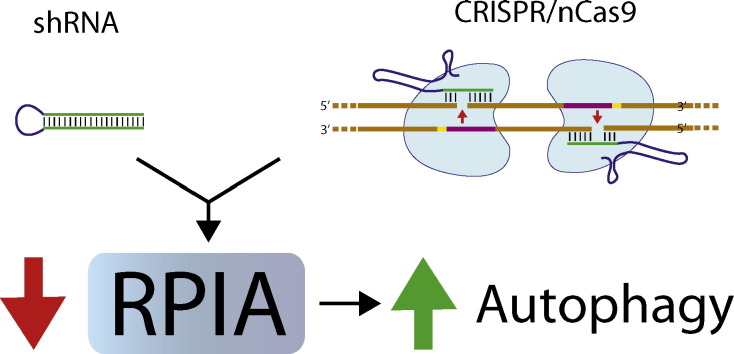

## 1 Introduction

Macroautophagy (hereafter autophagy) is a physiological process in response to low levels of metabolites and nutrients, that provides additional energy to the cell through the induction of a lysosomal degradation pathway and recycling of cellular constituents [31]. Conditions such as starvation, stress and pathogen infections induce autophagy, and basal autophagy is an important process for cellular homeostasis. A key regulator of autophagy is the serine/threonine kinase mTORC1 (hereafter mTOR), which inhibits formation of autophagosomes. Inhibition of mTOR by knockdown or treatment with the small molecule inhibitors rapamycin or torin induces autophagy, and inhibition of upstream kinases such as AKT1 that activate mTOR also results in the induction of autophagy [8]. In the process of autophagosome maturation, members of the LC3 family are cleaved by ATG4 proteases, lipidated and incorporated into maturing autophagosomes, in which LC3B (hereafter LC3) is the predominant form. In this process, cytoplasmic LC3-I accumulates in LC3-positive puncta following lipid modification of LC3 (LC3-II) and can be used as a marker for autophagosome numbers. The various stages of LC3 maturation can also be measured by immunoblotting.

In the past several years, metabolic inputs such as AMP/ATP levels through AMP kinase regulation [2], [12], [22] and amino acid sensing by the Rag GTPase and mTOR [11], [35] have been found to regulate autophagy. Other metabolic processes such as glycolysis, Acetyl-CoA synthesis and fatty acid oxidation have been found to play an important role in the regulation of autophagy as well [13], [33], [39]. Overall, it is thought that catabolic processes increase autophagic flux, whereas anabolic processes may reduce autophagy. The control of autophagy by metabolic processes can be classified into two major pathways: one is via direct sensing of the levels of metabolic intermediates as is the case for amino acids, and the other is through mechanisms that modulate the oxidative status of the cell. Reactive oxygen species and reductive equivalents are pivotal in the regulation of autophagy. In most cells, the generation of reductive equivalents is mediated by the pentose phosphate pathway (PPP).

The PPP catabolizes glucose-6-phosphate to generate reductive equivalents in the form of NADPH, and in addition, generates ribose-5-phosphate, a precursor for synthesis of pentose-ribosyl pyrophosphate (PRPP) and all nucleotides. Enzymes within this pathway, including glucose-6-phosphate dehydrogenase (G6PD) and transketolase have been proposed as tumor oncogenes [25], [26], suggesting that their control is de-regulated in certain cancers. In addition, regulatory control of ribose-phosphate pyrophosphokinase (PRS2) and G6PD by mitogenic signals can account for increased enzymatic activities under certain conditions [4], [16], [34], [40], [41], [44], [45]. Starvation results in drastic changes in the activity of enzymes in this pathway, leading to decreased PRPP synthesis and increased production of xylulose-5-phosphate [5], [6], [36]. The sensitivity to growth conditions and implication in some forms of tumor development suggest that enzymes in the pentose-phosphate pathways are responsive to, and perhaps control, cell survival pathways, mitogenic signaling, and autophagy.

Ribose-5-phosphate isomerase (RPIA) catalyzes the first step of the non-oxidative branch of the PPP and as such is positioned to act as the gatekeeper that determines whether the pathway primarily generates PRPP and nucleotide precursors, or alternatively recycles metabolites for generation of NADPH and reductive equivalents. Both processes have previously been linked to autophagy. This report identifies the consequences of RPIA depletion to autophagy. We have applied a rigorous approach applying a combination of shRNA-mediated knockdown and CRISPR/Cas9 genome engineering to validate the effect of RPIA depletion on autophagosome formation.

## 2 Materials and methods

### 2.1 Plasmids

The pX335 CRISPR/nCas9 plasmid was obtained from Addgene (#42335). Oligos used for cloning are described in Table 1. pLKO.1 control and RPIA shRNA plasmids were purchased from Sigma (see Table 1). The retroviral shRNA hairpin vector pMOWS-5.2 was generated by inserting the full H1 promoter sequence into pMOWS [18]. The shRNA sequences were cloned as adapters in pMOWS-5.2 immediately upstream of the H1 promoter using *Eco*RI and *Bam*HI. The empty pMOWS-5.2 vector or a vector with shRNA targeting GFP were used as controls. RPIA was sub-cloned using the *Eco*RI and *Not*I restriction sites in pEAK12-Flag. Primers used are indicated in Table 1.

### 2.2 Cell culture

All cell lines were cultured in Dulbecco's modified Eagle's medium (DMEM), + high glucose, + GlutaMAX™ (ThermoFisher Scientific®, 61965-026) and supplemented with 1 mM sodium pyruvate (ThermoFisher Scientific®, 11360-070), 100 U/ml Penicillin-Streptomycin (ThermoFisher Scientific®, 15140-122) and 10% Fetal Calf Serum (Sigma, 12133C) unless otherwise stated. All live cells were incubated at 37 °C and 5% CO2. Murine fetal liver cells from day 13.5 embryos were prepared as described [19]. Following compounds/reagents were used as indicated: bafilomycin A (Sigma®, B1793), puromycin dihydrochloride (Sigma®, P9620), DMSO (Sigma®, D2650) Earle's Balanced Salt Solution (EBSS; ThermoFisher Scientific®, 24010-043), torin 1 (Merck-Millipore, 475991) and *N*-acetyl cysteine (Sigma, A9165).

### 2.3 Transfection and transduction

Transfection and transduction were performed as previously described [21]. Cells were seeded at appropriate densities (to achieve 20–50% confluence on the following day) in 6 or 12 well plates for western blotting and in 96 well plates for imaging and luciferase assays. On the following day, cells were transiently transfected with 1 μg (6 well), 0.5 μg (12 well) or 100 ng (96 well) of control and knockdown/overexpression plasmids as indicated using Xtreme gene 9 (Roche) or polyethylenimine (PEI) for 6/12 wells and lipofectamine 2000 (Thermo Fisher Scientific) or PEI for 96 wells according to the manufacturers' instructions.

### 2.4 Western-blotting

Total protein of the cell lysate was extracted from cells (after treatment as indicated) in confluent 6 or 12 well dishes after 1 × wash with PBS by using 60–100 μl of Nonidet P-40 (NP-40) lysis buffer (50 mM Tris-HCl pH 7.4, 200 mM NaCl, 0.1 mM EDTA, 10% glycerol, 0.5% NP-40) including cOmplete and PhosSTOP protease & phosphatase inhibitors (Roche, 4906837001 & 11873580001). The cells were incubated with lysis buffer for 10 min, then transferred to centrifuge tubes and centrifuged at 13,200 rpm for 20 min at 4 °C. The supernatant was then transferred to a new tube. Protein concentration was determined with a BCA assay kit (Thermo Scientific, #23228) according to the manufacturer's instructions. Equal amounts of protein (15–20 μg) for each sample were mixed with 2 × Laemmli sample buffer (4% SDS, 10% 2-mercaptoethanol, 20% glycerol, 0.004% bromophenol blue, 0.125 M Tris-HCl pH 6.8), heated at 95 °C for 5 min, separated on a 4–20% SDS–PAGE gel and then transferred to a PDVF membrane (Millipore, #1620260). After transfer, presence of proteins and equal loading of lysates was confirmed using Ponceau S (Sigma, P3504) and membranes were blocked at 1 h in 5% milk powder in PBS-tween or 2% BSA in TBS-tween. The primary antibodies include anti LC3B (Sigma, L7543), anti phospho-S6 Kinase (Cell Signaling, 9234), anti S6 Kinase (Cell Signaling, 2708), anti p62/SQSTM1 (Sigma, P0067), anti Vinculin (Abcam, ab129002) and anti Actin (Sigma, 2228). Secondary antibodies used were IRdye 680RD and 800CW (Licor) or anti-rabbit HRP and anti-mouse HRP. Membrane was stripped when appropriate by using stripping buffer (62.5 mM Tris-HCL pH 6.8, 2% SDS and 100 mM β-mercaptoethanol). Bands were detected using an Odyssey fluorescence analyzer (Licor) or Image Quant (GE Healthcare Life Sciences). Densitometry analysis was performed using Fiji.

### 2.5 Luciferase assays

Luciferase assays were performed as previously described [27]. Briefly, supernatants were collected and mixed with substrate buffer containing native coelenterazine before reading in the PerkinElmer Envision II.

### 2.6 Immunofluorescence

Cells were washed 1 × with PBS, fixed for 15 min with 4% PFA at room temperature or with 100% cold methanol on ice (LC3-staining). For immunostaining, cells were washed 3 ×, permeabilized at room temperature with 0.1% TX-100 for 10 min and washed again 1 ×. Non-specific binding was blocked using 3% goat serum (Gibco) in PBS (blocking solution) for 30 min and the cells were then incubated with 1:200 diluted rabbit anti LC3B (Sigma, L7543) in blocking solution for 2 h. After washing 2 ×, cells were incubated with 1:400 diluted goat anti rabbit Alexa 568 (ThermoFisher Scientific, A-11011) for 1 h. After washing 2 ×, cells were stained with 1:10,000 diluted Hoechst 33342 (ThermoFisher Scientific, H1399) for 10 min and washed once.

### 2.7 Image acquisition and analysis

Images were acquired using an inverted confocal microscope (Leica TCS SPE) or an Opera LX microscope (Perkin-Elmer) with 6–12 replicas per condition and 30 images per well. Image analysis was performed using Fiji or Columbus (PerkinElmer). Briefly, cell nuclei (cell numbers) were identified and counted based on selection of the nuclear channel (405 nm) and nuclei detection method C. The cytoplasm was identified as above using method A (GFP intensity for knockdown, LC3 staining for CRISPR). Transfected cells were selected based on intensity threshold of the GFP channel (488 nm) and cell size. On those cells, spots were detected using the LC3 channel (562 nm) and spot detection method A, which is based on a threshold of relative spot intensity in the surrounding pixels. The selection criteria were further refined to select for puncta size and roundness to reduce the number of false positives. Puncta numbers were normalized to cell number and cell area, and puncta area was normalized to cell area. On Fiji, cell area (cytoplasm) was measured based on a binary threshold across all images & conditions. Puncta were measured as maxima with a noise threshold = 50.

### 2.8 RT-PCR

Total RNA was extracted using the GeneJET RNA Purification Kit (Fermentas) followed by DNase treatment (Fermentas). cDNA was synthesized with the RevertAid Reverse Transcriptase Kit (Fermentas) according to the manufacturer's instructions and using oligo(dT) primers. RPIA expression was measured with Maxima POWER SYBR Green Master Mix (Fermentas) in a CFx Connect optics module (BioRad) using GAPDH as reference gene. Primers used are indicated in Table 1. Relative RPIA transcriptional levels are presented as fold of 2^− ΔΔCt^ = 2^−(ΔCt RPIA − ΔCt GAPDH)^.

### 2.9 CRISPR/Cas9 genome editing

For genomic modification at the *RPIA* locus on chromosome 2p11.2 in HeLa cells, the CRISPR/Cas9 double nicking strategy [38] was applied as described. In brief, RPIA specific sgRNAs vectors targeting exon 1 were created and used according to the protocol. Oligo sequences that were used for sgRNA generation to clone into pX335 (containing nCas9) are indicated in Table 1. 6.5 × 10^4^ cells were seeded in 24 well plates and co-transfected the next day with 200 ng of each sgRNA vector (400 ng pX335 for control, labeled CR-WT) + 100 ng pBabe mCherry-puro (empty pMOWS for selection control) using lipofectamine according to the manufacturer's instructions. After 48 h, cells were selected in culture medium containing puromycin at 1.0 μg/ml and fresh selection medium was provided every 2–3 days. Cells were selected and expanded for a total of 18 days. Selection control cells were completely dead after 7 days. After 10 days, individual clonal colonies were picked using 3 mm trypsin-soaked cloning discs and further expanded in 24 well, 6 well and ultimately in 10 cm petri dishes. Genomic DNA was obtained using QuickExtract™ DNA Extraction Solution (Cambio, QE09050) according to the manufacturer's instructions and genomic PCR was performed using Pfusion polymerase (NEB) and primers as indicated in Table 1. Genomic PCR products were purified using the PCR cleanup kit (Qiagen) and run on a 2% Agarose gel, or directly sequenced (Source BioScience's Sanger sequencing), or first cloned into pGEM-T easy (Promega) vectors prior to sequencing in order to identify the allelic variations on both chromosomes.

## 3 Results

### 3.1 RPIA inhibits ATG4B-mediated processing of LC3

**Fig. 1:**
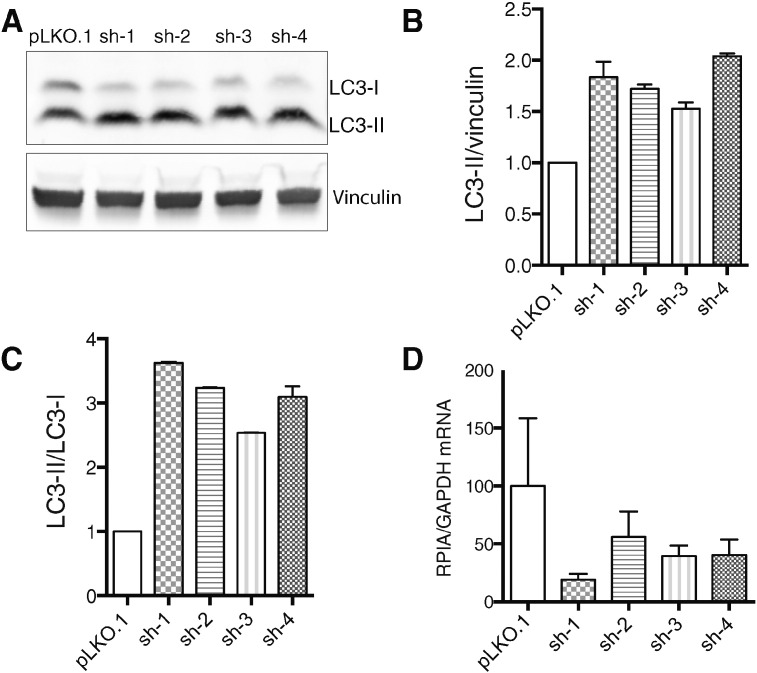
LC3-processing is increased upon knockdown of RPIA. A) Immunoblot of LC3-processing in HeLa cells at 72 h post-transfection with control (pLKO.1) vector or shRNA vectors against RPIA (sh1–4). B, C) Densitometry analysis of LC-II/vinculin and LC3-II/LC3-I levels was performed using Fiji. Data represent mean ± SD, n = 2. D) Expression levels of RPIA at 72 h post-transfection in HeLa cells using qPCR, normalized to GAPDH. Data represent mean ± SD, n = 3.

In order to study the effect of RPIA depletion on autophagy, we tested four different shRNA targeting sequences for knockdown of human RPIA in HeLa cells. We monitored LC3 processing by immunoblotting, taking the increase in lipidated LC3-II, which correlates with increased autophagosome numbers, as a surrogate for the induction of the autophagic state. Upon transfection of shRNAs targeting RPIA, we observed a significant increase in LC3-II compared to LC3-I, suggesting an increase in basal autophagy (Fig. 1A), with all four shRNA sequences being equally effective to cause an increase in LC3-II to LC3-I and LC3-II to loading control (vinculin) ratios (Fig. 1B, C). Efficient knockdown of RPIA transcripts by all four shRNAs was shown by RT-PCR (Fig. 1D).

**Fig. 2:**
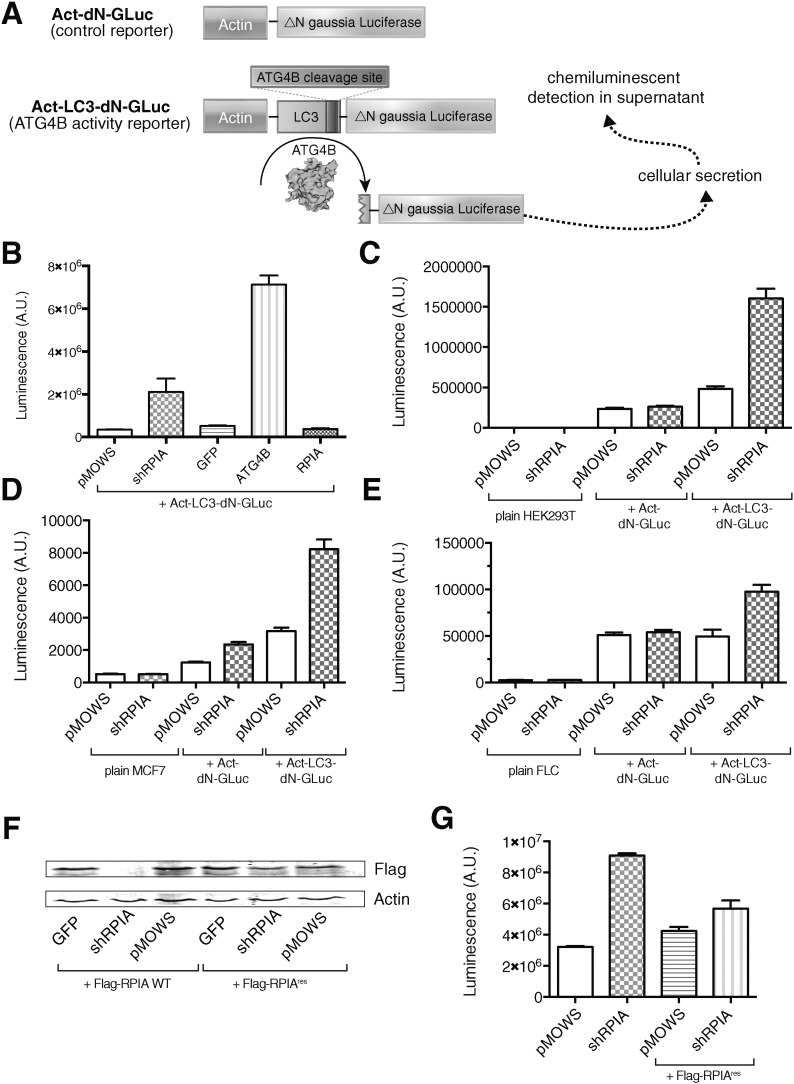
Knockdown of RPIA increases ATG4B-mediated cleavage of LC3. A) Schematic overview of the ATG4B-mediated luciferase release assay. Luciferase is secreted from the cells and a chemiluminescence signal in the supernatant can be measured. B) Luciferase release assay 24 h post-transfection with the indicated plasmids in 293ET cells transduced with the indicated reporter constructs. Data represent mean ± SD, n = 3. C) Luciferase activity of supernatant collected 96 h post-transduction with control (pMOWS) and shRPIA in 293ET cells as indicated. Data represent mean ± SD, n = 3. D, E) Luciferase activity of supernatants in transduced MCF7 (E) and primary murine fetal liver cells (F) collected 96 or 24 h hours post-transduction with control (pMOWS) and shRPIA, respectively. Data represent mean ± SD, n = 3. F) 293ET cells were co-transfected with Flag-RPIA-WT and Flag-RPIA^res^ (resistant to shRNA knockdown) together with the indicated shRNA constructs. Cell lysates were resolved by PAGE and blotted using a Flag-antibody (top panel) and beta-actin as a loading control (bottom panel). These results demonstrate that RPIA^res^ is resistant to knockdown with shRPIA. G) Luciferase release assay 96 h post-transfection with control (pMOWS), shRPIA and ± shRNA resistant RPIA in 293ET cells expressing Act-LC3-dNGLuc. Data represent mean ± SD, n = 3.

Next, we investigated whether the effect of RPIA depletion on LC3 processing is via the autophagy protease ATG4B. By using the *Gaussia* luciferase release assay to monitor cellular ATG4B activity (Fig. 2A) as previously described [17], [20], we assessed the effect of shRNA-mediated knockdown of RPIA on ATG4B activity. The sh-4 sequence was inserted in the retroviral expression vector pMOWS-5.2, previously found to express transcripts at very high levels in a wide variety of cell types, including hematopoietic stem cells [18]. The construct (designated shRPIA) was co-transfected with Act-LC3-dNGLuc in 293ET cells and luciferase activity measured in supernatants after 24 h as readout for cellular ATG4B activity. Following the co-transfection of ATG4B cDNA as a positive control, we observed a 13.6-fold increase in luciferase activity in supernatants compared to control cells that were transfected with GFP cDNA (Fig. 2B). shRNA-mediated knockdown of RPIA gave a significant 4-fold induction of secreted luciferase compared to control cells (Fig. 2B). An increase in luciferase release was not observed in cells expressing an Act-dNGLuc construct that is devoid of the LC3 cleavage motif (Fig. 2C), indicating that shRPIA specifically enhances cleavage of LC3. These results were confirmed in MCF7 cells (Fig. 2D) and fetal liver cells, which express high levels of RPIA (Fig. 2E).

Next, we generated a Flag-tagged mutant RPIA cDNA sequence with silent nucleotide changes (designated Flag-RPIA^res^) that confers resistance to shRPIA-mediated knockdown. shRPIA-mediated knockdown significantly reduced the levels of wild-type RPIA (Flag-RPIA-WT), but did not affect the levels of Flag-RPIA^res^ (Fig. 2F), validating its use for rescue experiments. When Act-LC3-dNGLUC was co-transfected with shRPIA, an increase in ATG4B activity was observed (Fig. 2G). Upon co-transfection of Flag-RPIA^res^, this increase in released luciferase by shRPIA was reverted, thus attesting to the on-target activity of shRPIA (Fig. 2G). These results support the notion that shRNA-mediated knockdown of RPIA impairs ATG4B-mediated LC3 processing.

### 3.2 RPIA knockdown increases autophagosome numbers

**Fig. 3:**
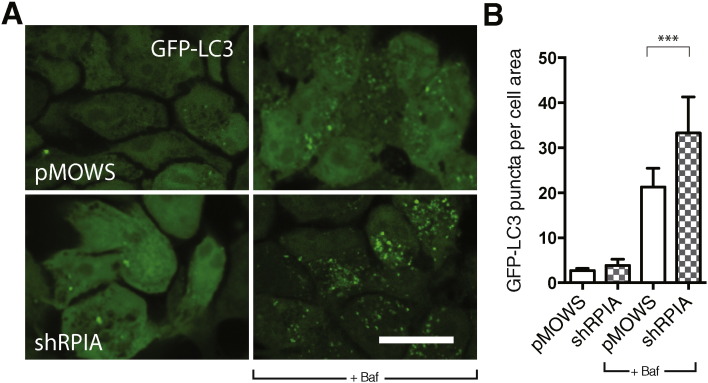
GFP-LC3 puncta are increased upon knockdown of RPIA. A) GFP-LC3 puncta in stably expressing GFP-LC3 cells, transfected with control (pMOWS) and pMOWS-shRPIA, imaged after 96 h knockdown. Cells were treated 1% DMSO or with 10 nM bafilomycin A (baf) in EBSS medium for two hours. Images were acquired on an inverted confocal Leica SPE microscope with a 63 × objective, scale bar = 10 μm. B) Puncta were identified and analyzed using Fiji image analysis software. Data represent mean ± SD of 1000–3000 cells from 2 independent experiments. ***p < 0.001.

Next, we wanted to test whether an increase in ATG4B-mediated LC3 processing is accompanied by an increase in autophagosome numbers. We transfected shRPIA or control vector in stably expressing GFP-LC3 HeLa cells, treated cells with bafilomycin A or DMSO control and observed GFP-LC3 puncta formation by fluorescence microscopy. We observed an increase in LC3 puncta under basal conditions that was further enhanced upon treatment with bafilomycin (Fig. 3A, B). These results suggest that RPIA suppresses basal autophagy. Following prolonged culture, the cells developed a weaker phenotype, suggestive of an adaptation to RPIA deficiency, an effect that has been observed for other metabolic enzymes [30].

### 3.3 Generation of a CRISPR knockout cell line deficient in RPIA

**Fig. 4:**
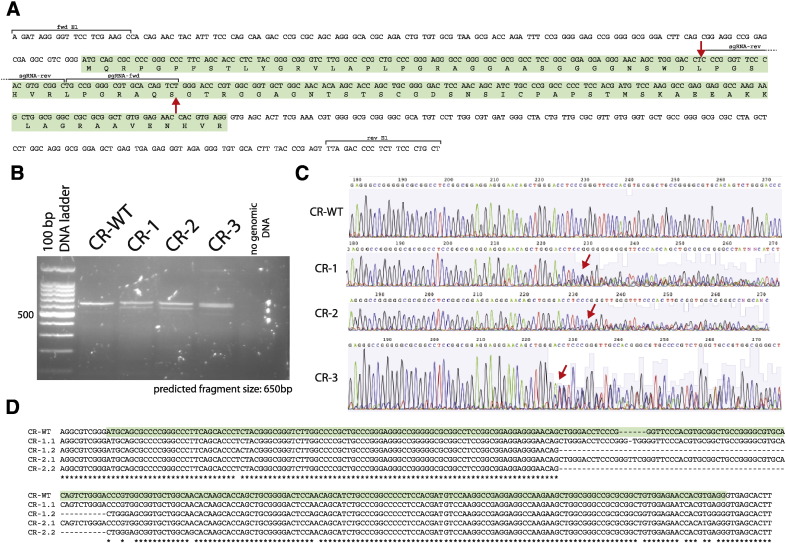
CRISPR cell line generation. A) Genomic region of RPIA Exon 1 on chromosome 2p11.2, showing the exon (green box), sgRNAs (fwd/rev) and genomic primers (E1 fwd/rev) used in B-D. Red arrows show the predicted nicks. B) Genomic amplification of control (CR-WT) and CRISPR-RPIA clones (CR1–3) on a 2% agarose gel. C) Sequencing reaction results of CR-WT and CR clones by Sanger sequencing. Red arrows indicate start of mixed base pair reads. D) Sequence alignment of CR-WT and CR clones sequencing reactions using ClustalW.

To further validate the shRNA findings, we modified the RPIA locus in HeLa cells using CRISPR/Cas9 genome editing [14]. In order to reduce potential off-target effects, we used a double nickase approach [38], designing two guide RNA (gRNA) sequences that target the first exon of RPIA, spaced 40 nucleotides apart (Fig. 4A). It has previously been shown that introduction of staggered nicks into genomic DNA frequently leads to generation of a double-stranded break (DSB), which is imperfectly repaired by non-homologous-end-joining (NHEJ), creating insertions/deletions (Indels) that disrupt gene function. RPIA-modified cell lines (labeled CR1–3) were generated by co-transfection of two sgRNAs + nCas9 and a pBABE-mCherry/puromycin containing vector, and a control cell line (CR-WT) was also generated using the same protocol but with a vector lacking the sgRNA sequences. Successful modification in puromycin-selected and expanded clones was initially tested by genomic PCR amplification (Fig. 4B). Three clones that showed a modification at the predicted locus and control cells were then subjected to Sanger sequencing of PCR amplicons. In all three clones (CR1–3), only mixed sequence traces were observed at the expected location, indicating that the genomic sequence had been modified (Fig. 4C). A large-scale mapping of the HeLa cell genome has recently identified that HeLa cells have a copy number of 2 or less for the majority of chromosome 2 [1], including the RPIA locus. Therefore, we expect to recover two different allelic mutations within the genome-edited region. In order to identify the exact genomic modification on both chromosomes at this locus, we sub-cloned the genomic PCR products and sequenced individual bacterial clones (Fig. 4D). From all three RPIA-modified clones, we did not recover any wild-type sequence. From clone CR‐1, all sequenced plasmid inserts showed out-of-frame mutations, whereas plasmids from clones CR–2 and CR–3 showed at least one in-frame mutation in addition to out-of-frame mutations. From clone CR–2, one insert sequence with a 6 bp insertion was recovered, whereas from CR–3, a 12 bp deletion was identified. For all three clones, 2 non-identical modified alleles were identified. Based on the observed sequence changes, transcription of RPIA in clone CR–1 is expected to produce a missense product from both alleles that is deficient in full-length RPIA.

**Fig. 5:**
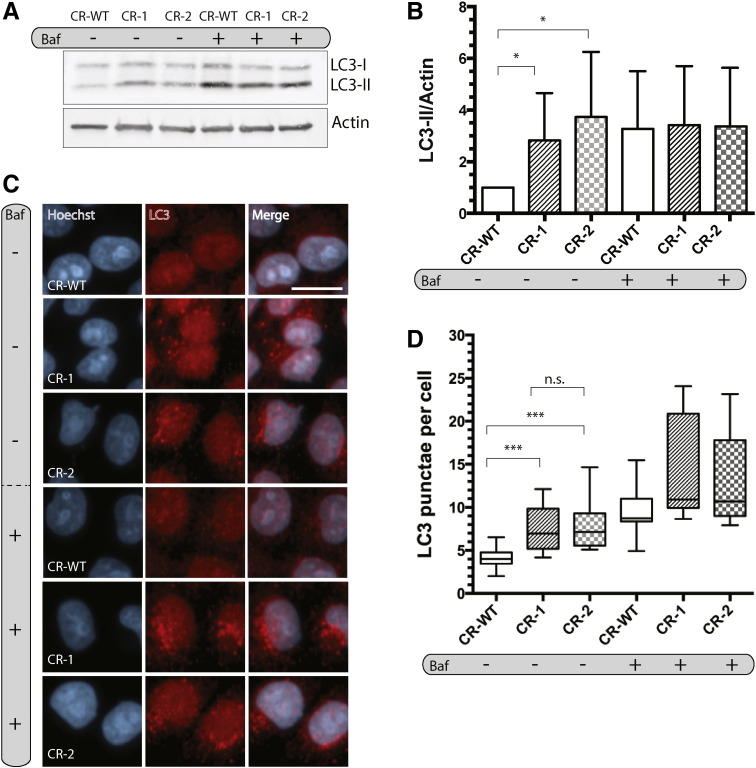
LC3-II levels and LC3 puncta are increased in CRISPR cell lines. A) Immunoblot of LC3-processing in control cells (CR-WT), CR-1 and CR-2, treated with 10 nM bafilomycin A (baf) for 2 h. B) Densitometry analysis of relative LC-II/Actin levels using Fiji. Data show the mean ± SD, n = 5. *, p < 0.05. C) Endogenous LC3 puncta in fixed CR-WT, CR-1 and CR-2 that were treated with 10 nM baf for 2 h. Images were acquired on an Opera LX microscope, scale bar = 10 μm. D) Quantification of C) using Columbus image analysis software. 10,000–28,000 cells per condition were analyzed in 3 independent experiments. ***, p < 0.001; n.s. = non-significant.

We then used CR–1 and CR–2 to assess the effect of RPIA knockout on LC3 processing and LC3- positive puncta. Under basal conditions, we observed an increase in the ratio of LC3-II to the loading control by immunoblotting, but not following treatment with bafilomycin A, an inhibitor of lysosome acidification, when compared to CR-WT control cells (Fig. 5A–B). Similarly, both mutant clones showed a remarkable increase in LC3-positive puncta compared to CR-WT cells (Fig. 5C–D). Upon treatment with bafilomycin A, an increase in LC3-positive puncta was observed for control cells, that was not observed in clones CR–1 and CR–2 (Fig. 5C-D). Thus, our results confirm that RPIA is an endogenous inhibitor of basal autophagy.

### 3.4 Enhanced LC3 processing upon knockdown of RPIA can be reversed by treatment with the antioxidant *N*-acetyl cysteine

**Fig. 6:**
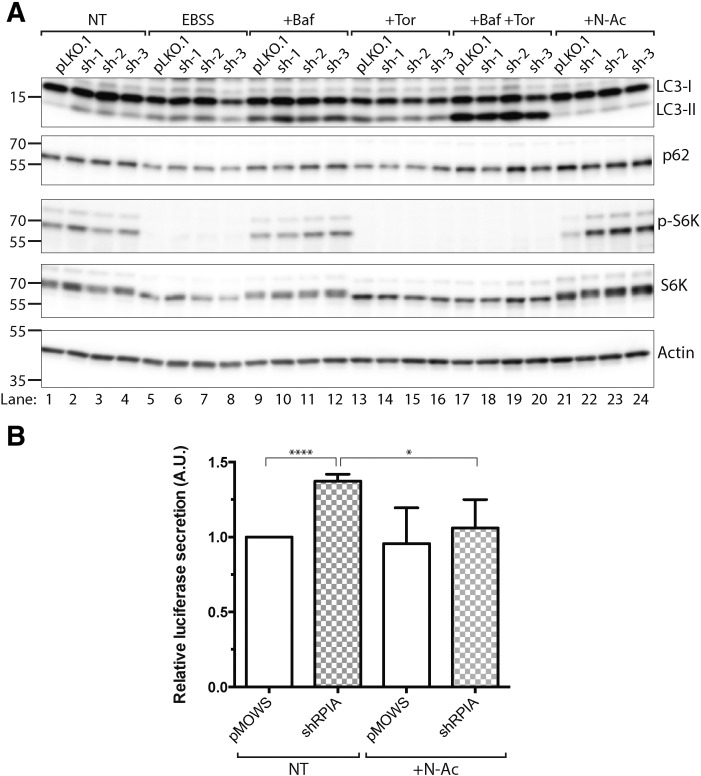
*N*-Acetyl cysteine reverts the effect of RPIA knockdown on LC3 processing. A) Immunoblot of LC3-processing and S6 Kinase signaling pathway in 293 cells transfected with pLKO.1 control and shRPIA (sh1–3) for 72 h, treated with EBSS, 10 nM bafilomycin A, 250 nM torin 1, 10 nM bafilomycin A + 250 nM torin1 and 1 mM *N*-acetyl cysteine for 3 h before harvesting. NT – not treated, EBSS - Earle's Balanced Salt Solution, Baf – bafilomycin, Tor – torin 1, and N-Ac – *N*-acetyl cysteine. B) Luciferase activity of supernatant collected 48 h post-transfection of control (pMOWS) and shRPIA, in 293 T cells expressing Act-LC3-dNGLuc. Cells were treated overnight with *N*-acetyl cysteine (10 mM). Data represent mean ± SD, n = 3.

In order to gain mechanistic insights into how RPIA regulates LC3 processing and autophagy, we investigated the consequence of RPIA knockdown on metabolic signaling pathways. We transfected shRNA vectors targeting RPIA that significantly reduced RPIA transcript levels (Fig. 1D) in HEK293T cells and performed immunoblotting for LC3, p62, phospho-S6 kinase and total S6 kinase. Cells were treated with torin to block mTOR signaling, bafilomycin A to disrupt autophagic flux, a combination of torin plus bafilomycin A, and *N*-acetyl cysteine to modulate the redox state of cells. Further, cells were starved in EBSS medium (Fig. 6). As previously observed (Figs. 1A, 5A), depletion of RPIA under basal conditions enhanced the level of LC3-II (Fig. 6A, lanes 1–4). Treatments with EBSS, bafilomycin, torin and bafilomycin plus torin resulted in a significant increase of LC3-II levels, in line with their expected action to enhance processing of LC3 (Fig. 6A, lanes 1, 5, 9, 13, 17). Knockdown of RPIA did not significantly further increase LC3-II levels upon these treatments, in line with our previous observations. p62 levels were reduced upon treatment with EBSS and torin, an effect that was reversed by co-treatment of torin with bafilomycin A (Fig. 6A). Knockdown of RPIA under any of these conditions did not affect p62 levels. Similarly, phospho-S6K levels were not changed by RPIA depletion, while phospho-S6K was sensitive to mTOR inhibition by EBSS, torin and torin plus bafilomycin A. Overall, these results suggest that RPIA-mediated inhibition of LC3 processing does not correlate with changes in mTOR-mediated signaling.

Next, in order to investigate whether RPIA-mediated inhibition of LC3 processing is sensitive to treatment with an antioxidant agent, we treated cells with *N*-acetyl cysteine and monitored LC3 processing and signaling responses. Interestingly, the increase in LC3-II under basal conditions upon knockdown of RPIA was completely reversed by treatment with *N*-acetyl cysteine (Fig. 6A, lanes 17–20). Similarly, enhanced luciferase release upon RPIA knockdown under basal conditions was reversed by treatment with *N*-acetyl cysteine (Fig. 6B), supporting the notion that the observed effects involve redox signaling responses.

Collectively, these data are consistent with the view that RPIA suppresses autophagosome formation and impairs LC3 processing.

## 4 Discussion

The interplay between autophagy, metabolism and signaling is not very well understood. The regulation of autophagy by the metabolic state of cells is an emerging field of research, and recently multiple metabolic enzymes have been implicated in the regulation of autophagy, including transketolase [32], fatty acid synthase [42], phosphofructokinase [24], [47], transglutaminase [3], [9], [10], [43] and acetyl-coenzyme A synthase [13], [29]. These studies suggest that additional metabolic pathways influence the regulation of autophagy, in addition to amino acid sensing by Rag GTPase/mTOR, and AMP kinase mediated signaling to suppress ULK1 activity in early steps of autophagosome formation [12], [22]. Here, we have identified a gene in the pentose phosphate pathway that inhibits autophagosome formation and LC3 processing.

There is some evidence that RPIA is involved in a number of diseases, including metabolic disorders and cancer. Mutations in the RPIA gene cause a rare disease called ribose-5-phosphate isomerase deficiency [15]. One patient has been identified with this disorder that manifests with leucoencephalopathy and peripheral neuropathy. The role of RPIA in other diseases requires further investigation. For instance, while compelling arguments have been made for metabolic products of the PPP in cancer cell regulation, it is possible that RPIA exerts non-enzymatic signaling functions that may contribute to disease. Furthermore, a recent study pointed out that modulating RPIA levels might serve as therapeutic strategy for aging and neurodegenerative disorders [46].

Recently, a role for RPIA in oncogenic signaling has been identified. A link between RPIA and PP2A/ERK signaling in hepatocarcinogenesis was reported [7] and it was found that RPIA mRNA expression levels are increased in HCC patients. Cells overexpressing RPIA showed increased proliferation, enhanced colony formation and accelerated tumor growth in a xenograft mouse model. siRNA-mediated knockdown of RPIA resulted in reduced cell growth in Hep3B and PLC5 liver cancer cell lines. However, RPIA may play an inhibitory role in the progression of some tumors, as increased hyper methylation of the RPIA locus has been observed in breast cancer [23]. Furthermore, it has been shown that microRNAs can target the RPIA gene and the downstream PRPS1 gene that drives nucleoside formation from ribose-5-phosphate, leading to reduced PPP flux and proliferation in human colorectal cancer cells [37]. PRPS1 regulates the formation of PRPP from ribose-5-phosphate and ATP. Thus, the PRPS1/2 genes are key molecules for diverting the PPP metabolites into the nucleoside formation branch. Indeed, many diseases have been linked to PRPS1/2, including cancer. How the observed role in oncogenic signaling is linked to autophagy remains to be studied in more detail. Overall, we propose a role for RPIA in the regulation of autophagy, which will have importance for the selection of anti-cancer therapies targeting this novel mode of regulation.

## Conflict of interest

The authors declare no conflict of interest.

## Figures and Tables

**TABLE 1:** Sequences of various primers used in this study. F – forward and R – reverse, RT – reverse transcription.

Designation	Sequence	Used in
sh-1	CCGGCGGGTACACAAATGGAGTGAACTCGAGTTCACTCCATTTGTGTACCCGTTTTTG	Knockdown of RPIA
sh-2	CCGGGCTGATGAAGTAGATGCTGATCTCGAGATCAGCATCTACTTCATCAGCTTTTTG	Knockdown of RPIA
sh-3	CCGGGAATTGGAAGTGGTTCTACAACTCGAGTTGTAGAACCACTTCCAATTCTTTTTG	Knockdown of RPIA
sh-4	CCGGGAAGTGAATACAGCTATCAAACTCGAGTTTGATAGCTGTATTCACTTCTTTTTG	Knockdown of RPIA, pMOWS cloning
RT-RPIA F	GGCGGTGCTGGCAACACAAG	RT-PCR
RT-RPIA R	TGGCGGGCCTGGAAGGAAGT	RT-PCR
GAPDH F	GAAATCCCATCACCATCTTCCAGG	RT-PCR
GAPDH R	GAGCCCCAGCCTTCTCCATG	RT-PCR
sgRNA-R	CACCGGCCGCACGTGGGAACCCGGG	CRISPR reverse strand nicking
sgRNA-F	CACCGTGCCGGGGCGTGCACAGTCT	CRISPR forward strand nicking
CRISPR gen F	GCGAATCCAGATAGGGGTTCCTCGAAGC	CRISPR - genomic PCR
CRISPR gen R	GCAAGCTTAGCAGGGAAGAGGGGTCTAA	CRISPR - genomic PCR
